# Tumor-Associated Disialylated Glycosphingolipid Antigen-Revealing Antibodies Found in Melanoma Patients' Immunoglobulin Repertoire Suggest a Two-Direction Regulation Mechanism Between Immune B Cells and the Tumor

**DOI:** 10.3389/fimmu.2019.00650

**Published:** 2019-04-05

**Authors:** Beatrix Kotlan, Szabolcs Horvath, Klara Eles, Vanda K. Plotar, Gyorgy Naszados, Katalin Czirbesz, Miri Blank, Emil Farkas, Laszlo Toth, Jozsef Tovari, Andras Szekacs, Yehuda Shoenfeld, Maria Godeny, Miklos Kasler, Gabriella Liszkay

**Affiliations:** ^1^Molecular Immunology and Toxicology, National Institute of Oncology, Budapest, Hungary; ^2^Center of Surgical and Molecular Pathology, National Institute of Oncology, Budapest, Hungary; ^3^Center of Image Analysis and Radiological Diagnostics, National Institute of Oncology, Budapest, Hungary; ^4^Department of Oncodermatology, National Institute of Oncology, Budapest, Hungary; ^5^Zabludowitz Center for Autoimmune Diseases, Sheba Medical Center Affiliated to Sackler Faculty of Medicine, Tel-Aviv University, Tel Aviv, Israel; ^6^Center of Oncosurgery, National Institute of Oncology, Budapest, Hungary; ^7^Department of Experimental Pharmacology, National Institute of Oncology, Budapest, Hungary; ^8^Agro-Environmental Research Institute, National Agricultural Research and Innovation Centre, Budapest, Hungary; ^9^National Institute of Oncology, Budapest, Hungary; ^10^Ministry of Human Capacities, Budapest, Hungary

**Keywords:** B lymphocytes, glycosphingolipids (GSLs), immune regulation, immunoglobulin, metastatic melanomas, natural antibody, tumor-associated antigen

## Abstract

There is far less information available about the tumor infiltrating B (TIL-B) cells, than about the tumor infiltrating T cells. We focused on discovering the features and potential role of B lymphocytes in solid tumors. Our project aimed to develop innovative strategies to define cancer membrane structures. We chose two solid tumor types, with variable to considerable B cell infiltration. The strategy we set up with invasive breast carcinoma, showing medullary features, has been introduced and standardized in metastatic melanoma. After detecting B lymphocytes by immunohistochemistry, VH-JH, Vκ-Jκ immunoglobulin rearranged V region genes were amplified by RT-PCR, from TIL-B cDNA. Immunoglobulin variable-region genes of interest were cloned, sequenced, and subjected to a comparative DNA analysis. Single-chain variable (scFv) antibody construction was performed in selected cases to generate a scFv library and to test tumor binding capacity. DNA sequence analysis revealed an overrepresented VH3-1 cluster, represented both in the breast cancer and the melanoma TIL-B immunoglobulin repertoire. We observed that our previously defined anti GD3 ganglioside-binder antibody-variable region genes were present in melanoma as well. Our antibody fragments showed binding potential to disialylated glycosphingolipids (GD3 ganglioside) and their O acetylated forms on melanoma cancer cells. We conclude that our results have a considerable tumor immunological impact, as they reveal the power of TIL-B cells to recognize strong tumor-associated glycosphingolipid structures on melanomas and other solid tumors. As tumor-derived gangliosides affect immune cell functions and reduce the B lymphocytes' antibody production, we suspect an important B lymphocyte and cancer cell crosstalk mechanism. We not only described the isolation and specificity testing of the tumor infiltrating B cells, but also showed the TIL-B cells' highly tumor-associated GD3 ganglioside-revealing potential in melanomas. The present data help to identify new cancer-associated biomarkers that may serve for novel cancer diagnostics. The two-direction regulation mechanism between immune B cells and the tumor could eventually be developed into an innovative cancer treatment strategy.

## Introduction

Tumor immunological investigations have reached an immunotherapeutic breakthrough in cancer research and therapeutics (1, 2). However, new strategies are still needed to solve the cure of various cancer types. Earlier, cancer research was more focused on T cell-related immunological mechanisms (3). Later, it became evident that immune B cells are also essential components in the “anti-tumor battle” of the immune system. However, there is far less information available about B cells accumulated in the tumor [tumor infiltrating B (TIL-B) cells] (4, 5), than about T cells (tumor infiltrating T cells) (6, 7). This is probably because the very small amounts of TIL-B cells are hard to discover. More sophisticated detection and characterization methods are required to reveal the minute amounts of immune B cells in the cancerous tissue. New strategies, involving molecular immunological and biotechnological techniques, led to pioneering results on TIL-B cells in invasive breast carcinoma with medullary features (8, 9). Further studies in melanoma and some other solid tumors enlarged our knowledge on TIL-B cells (10–12). There are still unanswered questions: Why are TIL-B cells accumulated in the tumor? What are the main roles of TIL-B cells? What are the features of the TIL-B cell antibody repertoire? Do TIL-B cells have an essential impact on cancer cell recognition and elimination? The lack of interest and limited methodology have hindered the discovery within this important field until recently.

Tumor infiltrating B lymphocytes have been recognized as a new hallmark of breast cancer (13). Answering questions about TIL-B cells' functions remains a major challenge. To help address this issue, our previous study on breast carcinomas has been extended to malignant melanomas. We are convinced that systematic studies, various immunological and molecular-biological investigations and DNA sequence analysis have the power to reveal characteristics of cells hidden in minor quantities in the tumor microenvironment. Through the comparison of different cancers, we hope to gain insight into the way TIL-B cells perform specific tasks in the tumor microenvironment and to establish their relationship to cancer cells. We defined earlier essential TIL-B cell-targeted components in breast carcinomas, that is, the tumor-associated disialylated glycosphingolipids (GD3 gangliosides) (9). The difference in carbohydrate profiles between normal and cancerous tissues is a major issue. The characteristic carbohydrate expression associated with malignant transformation is caused by “aberrant glycosylation.” Complex carbohydrate cell membrane components are the glycoproteins, proteoglycans, and glycosphingolipids (14, 15).

Due to the importance of disialylated GSLs in cancer progression, it seemed obvious to detect these cancer-associated membrane structures on melanoma cells and tissue sections. Parallelly, we examined in detail the B cell infiltration and antibody profile in melanomas. We hypothesized a causal link between the TIL-B produced antibody repertoire and the extensive expression of highly tumor-associated glycosphingolipid membrane structures on the cell surface of highly malignant solid tumors. Our project with innovative technological strategies not only opens up for a deeper understanding of these key elements in cancer progression and metastases, but will help to build various types of new diagnostic possibilities linked to these glycolipid-based cancer membrane structures.

## Materials and Methods

We set up a complex strategy with immunologic, molecular genetics, and biotechnological methods and processed cancerous tissue specimen in order to approach the questions on tumor infiltrating B (TIL-B) cells.

### Cancerous Tissue

Cancerous tissue punch biopsy samples and peripheral blood of patients with melanoma were tested with ethical permissions (ETT TUKEB 16462-02/2010, 336/2014.9710-1/2015/EKU) received for our project. Previously stored minor tissue samples, from surgically removed melanomas and breast carcinomas, were investigated as well. Fresh melanoma samples were used to set up primary cultures and were fresh frozen for immunohistochemistry and molecular processes. Formalin-fixed paraffin-embedded melanoma tissue sections were made additionally.

### Cancer Cell Lines

Primary tumor cell cultures were developed from fresh melanoma tissue samples. The melanoma cell line (SK-Mel28, M24) was purchased from American Type Cell Collections (ATCC), A-2058 melanoma cell line was an earlier generous gift to the laboratory from professor Dr. Meenhard Herlyn and professor Dr. Lance A Liotta. Primary breast cancer cultures that were developed in the course of previous studies and MDA MB-231, ZR751 cell lines purchased from ATCC were investigated. Cells were maintained in steady-state culture conditions in RPMI-1640 culture media (Sigma, St. Louis, MO, USA) supplemented with 5% fetal bovine sera (FBS), penicillin/streptomycin) (1:100) (Sigma) and grown until confluent. To set up primary cell cultures, 0.8 μg/ml gentamicin and amphotericin B (70 μg/ml) (Sigma) were added to the media. Cells were grown in 25 or 75 cm^3^ tissue culture flasks (Greiner) under culture conditions (37°, humidified thermostat with 5% CO2). Growth rates and viability were followed by inverted and normal light microscopy (Olympos and Nikon, both from Shinjuku, Tokyo, Japan).

### Immunohistochemistry

Formalin-fixed and paraffin-embedded tissue sections were deparaffinized in Xilol and Ethanol. After this, hydrogen peroxidase blocking antigen retrieval was performed by heat-exposure in a Microwave (Meditest MFX800-3). Slides were blocked with 3% bovine serum albumin (BSA) in PBS and then reacted (4°C, overnight or 37°C, 30 min) with the monoclonal antibodies of interest. Tumor infiltrating B cells were detected by B cell-specific monoclonal antibodies (CD20) (DAKO). Novel disialylated glycosphingolipid-specific antibodies (GD3) (Calbiochem and Axxora/Alexis, Abcam, London, UK,), HCBC3 anti GD3 antibody (provided by Dr. Mark C Glassy) and our selected disialylated GSL-specific antibody fragments were tested. IHC was performed with Supersensitive TM One Step Polymer IHC Detection Kit System (BioGenex), using mainly ImPact TM AEC substrate (Vector).

### PCR Amplification of TIL-B Ig V Regions

Minor frozen tissue samples were homogenized under liquid nitrogen and stored in TRIZOL at −70°C until RNA was extracted according to the manufacturer's instructions (RNeasy Mini kit; Qiagen, Hilden, Germany and NucleoSpin RNA XS, Macherey-Nagel, Düren, Germany). cDNA was synthetized (Pharmacia Biotech kit) to amplify the human Ig VH-JH, Vκ-Jκ, and Vλ-Jλ encoding regions with specific primers designed previously (16). Polymerase chain reaction (PCR) was performed (35 cycles: 1 min, 94°C; 1 min, 60°C; and 1 min, 72°C) in a PerkinElmer/Cetus thermocycler.

### Cloning and Sequencing

Immunglobulin heavy and light chain variable region (VH-JH, Vκ-Jκ, and Vλ-Jλ) gene PCR products were purified, and then blunt end ligated into pUC18 *(Sma*I/BAP) plasmid vector (Pharmacia Biotech) before transformation into *Escherichia coli* TG1 bacteria. Gene-insert positive clones were selected by PCR screen technique (17). Sequencing of the plasmid dsDNA minipreps (QIAprep Spin kit; Qiagen) was performed by automatic sequencing (Dye Terminator Sequence Reaction Kit, DyeEx Spin kit (Qiagen; ABI PRISM Software, automatic sequencer of Perkin Elmer, and partly with commercially available sequencing service (Invitrogen, San Diego, CA).

### Comparative DNA Sequence Analysis

Comparative DNA sequence analysis was performed first using BIOEDIT (18), Clustal X 1.8 (19), and TREEVIEW 1.5.2 (20). In later work phases, we had the Vector NTI 11 available to make all the sequence homology analyses. For comparative DNA sequence analysis, we used KABAT National Institute of Health (http://immuno.bme.nwu.edu), New Kabat Database Server: george at immuno.bme.nwu.edu, and IMGT, the international ImMunoGeneTics database®, (www.imgt.org), (http://imgt.cines.fr) and (http://imgt.cnusc.fr:8104). Databank search via National Center for Biotechnology Information Blast server to GenBank/European Molecular Biology Laboratory Net databases was conducted to find homologous sequences and the generated data was termed as Blastn result.

### Construction of scFvK for Phage Library Generation

Assembly reactions of rearranged Ig V region H and L chain genes were conducted by a three-step PCR amplification, using a linker peptide (Gly4Ser3) coding sequence. Purified and suitable restriction enzyme digested VH-JK fragments were ligated into a phagemid vector (21), according to the methods we described earlier (17). However, slight modifications in terms of the library generation and panning process against membrane preparations were made. According to our previous antibody repertoire analysis in breast carcinomas, Vκ light chains were represented with a broader variability than Vλ light chains. Therefore, as a first choice, we were more interested in the Vκ representatives in melanoma.

### Soluble scFv Enzyme Labeled Immunosorbent Assay (ELISA)

Ninety-six-well Nunc MaxiSorp® flat-bottom plates were precoated (16 h, 4°C) with 1–10 μg of native tumor cell membrane preparations. Plates were washed repeatedly and blocked with 200 μl of 2% BSA in PBS. Soluble fractions of the test antibody fragments and control antibodies were incubated in triplicates for 16 h at 4°C. Detecting second antibody alkaline phosphate conjugated anti-c-myc (Sigma-Aldrich) and p-Nitrophenyl Phosphate (Sigma-Aldrich) substrate was used according to standard conditions. HCBC3 (anti GD3) and HCBD1 (anti GD2) monoclonal antibodies were Prof Dr. Mark C Glassy's generous gifts for testing.

### Immunofluorescence—Flow Cytometry, FACS Analysis

Melanoma cells were cultured until reaching confluence, harvested by EDTA with 0.02% PBS and incubated at 37°C for 30 min (or at 4°C overnight) with anti-ganglioside monoclonal antibodies (CVL-MAB0014-1) (Axxora, Farmingdale, NY, USA), MA1-25302 (Pierce, Thermoscientific, Rockford, IL, USA), AB13779 (Abcam, London, UK). Cancerous cell suspensions were reacted with unique GD3 ganglioside-specific antibodies (Abcam, London, UK), Calbiochem), HCBC3 anti GD3 antibody or soluble fractions of our expressed disialylated GSL binder antibody fragments. First and second antibody reactions were followed by wash steps with 1% BSA PBS and PBS. Anti-mouse (Fab')2 phycoerythrin (DAKO) or anti-mouse (Fab')2 FITC (Sigma) was used as second label antibody. Melanoma patient-derived primary cell suspensions of melanoma cells (SK-Mel 28, A-2058) were investigated by flow cytometry (CyFlow SL-Green, FloMax, Partec, Munster, Germany) and in some cases by FACSAvia Sorter/Beckton Dickinson. Forward and side scatter dot plots and immunohistological curves were evaluated for antigen expression intensity and the percentage of positive cells with data analyzing software Flo Max (Partec).

### Immunofluorescence—Confocal Laser Microscopy

Minor melanoma tissue samples were snap-frozen with isopentane in liquid nitrogen. Fresh frozen cancerous tissue cryostat sections [6–8 μm, freezing media (Bio-Optika, Milano, Slee Cryostat mnt (Auroscience)] of melanomas were fixed in 4% paraformaldehyde (PFA) PBS for 15 min. Three percent BSA PBS was used for blocking, before the monoclonal antibodies specific to tumor-associated disialylated glycosphingolipid antigens (Calbiochem, Abcam) were added for an overnight incubation at 4°C. Indirect immunofluorescence with FITC-labeled second antibodies (anti mouse IgG FITC from DAKO), or biotinylated rabbit anti mouse IgG (Fab')2 (1:100) and Streptavidin FITC (Vector Laboratories, Burlingame, CA, USA) with propidium-iodide (nuclear staining) was used. Chamber slides (Nunc Lab Tech) were used in certain cases to culture cancerous cells and IF label them *in situ*. Indirect immunofluorescence (FITC) labeling was detected in confocal laser microscopy (Nikon Eclipse E600, Nikon Model C1-Lu3, Tokyo, Japan) or conventional IF microscopy.

## Results

### Representative B Cell Antigenic Pattern Detected in Tissue Sections of Malignant Melanomas Enable the Investigations of TIL-B at a Molecular Level

**Figure 1:**
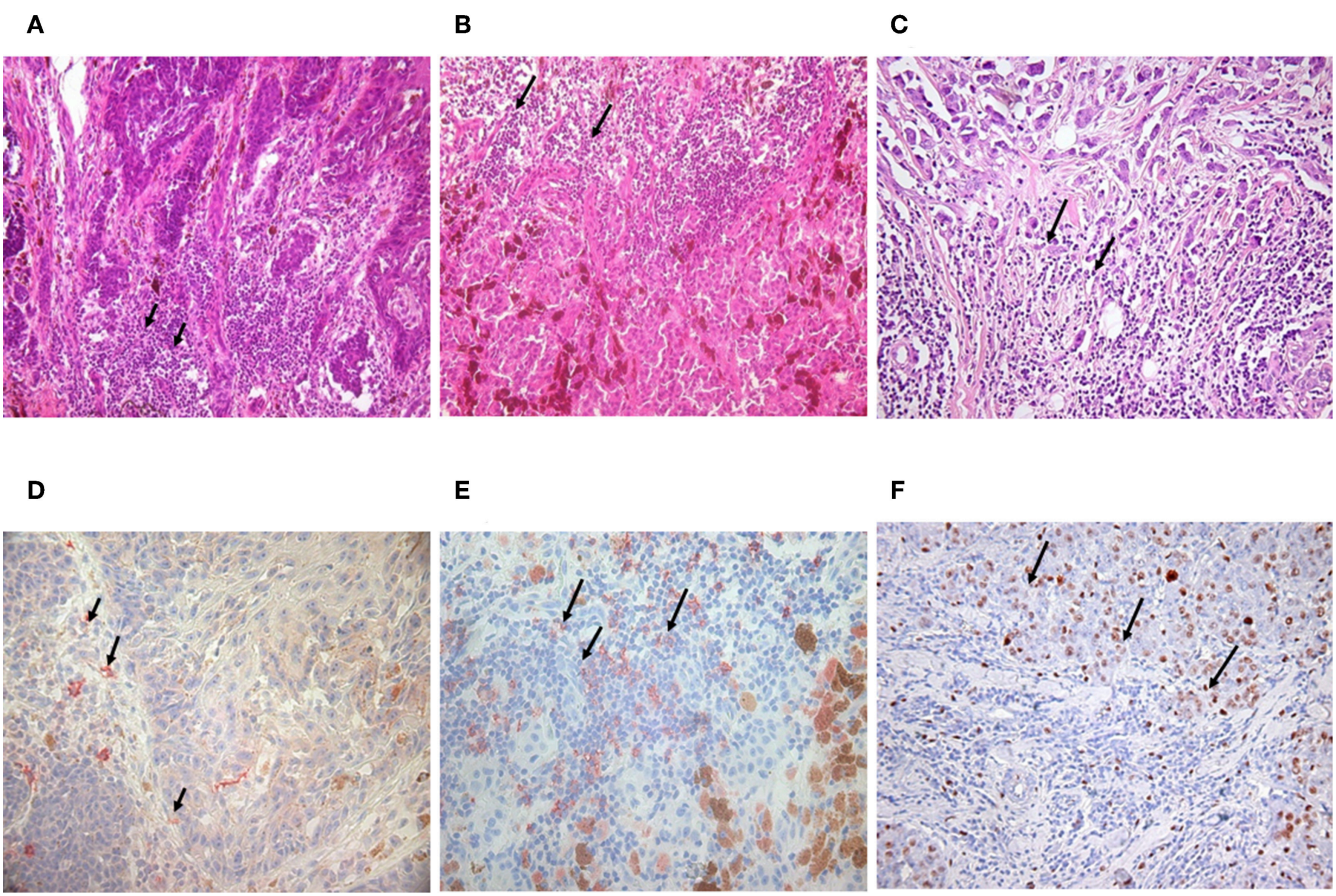
Detection of tumor infiltrating B lymphocytes in malignant melanoma and invasive ductal breast carcinoma. Immunohistology with haematoxilin & eosin staining showed the same abundant immune cell infiltration in nodular **(A)** and superficial spreading melanoma **(B)** as in invasive breast carcinoma with medullary features **(C)** black arrows. B lymphocytes could be defined with CD20 monoclonal antibody, One Step Polymer HRP detection system and AEC substrate in melanoma **(D,E)** and breast carcinoma by immunohistochemistry (100x). Black arrows are pointing to immune cell infiltration (immunohistology) or CD20 positive B cells labeled in red (immunohistochemistry) **(D,E)**. Investigations in breast carcinoma postulate that CD5 positive B cells are also present **(F)**.

**Figure 2:**
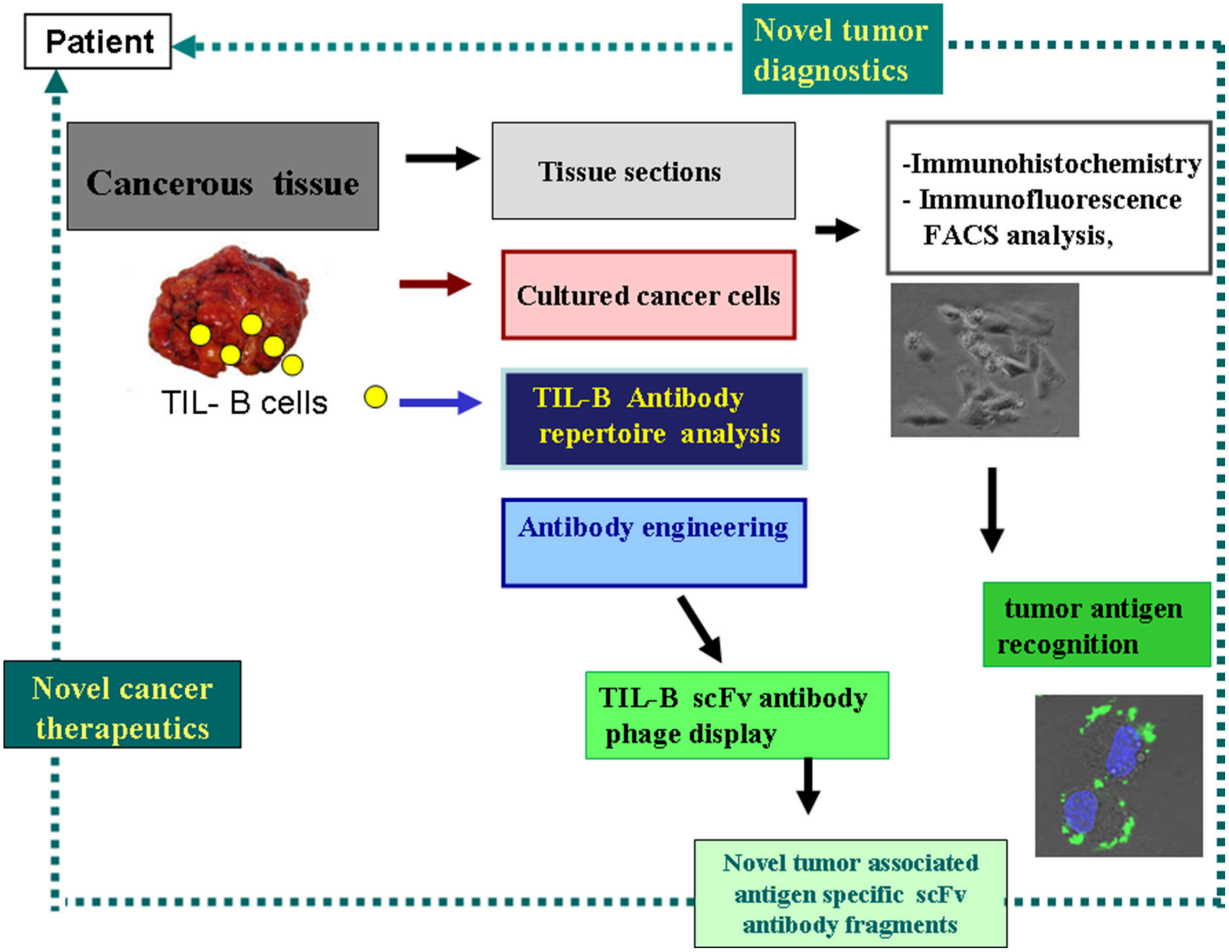
Strategy to harness the tumor infiltrating B cells' tumor antigen revealing capacity for diagnostics and therapeutics. This schematic flow chart clearly shows, how cancerous tissues containing immune B cells can be processed and investigated in the course of various immunological and molecular biological techniques. As a result of our strategy antibody fragments with tumor antigen specificity could be obtained, representing binding to cancer cells. Small picture (in lower-right corner) shows melanoma cells reacted with anti GD3 ganglioside antibody fragment in immunohistochemistry, with FITC labeled antibody and DAPI nucleus staining.

Malignant melanoma tissue sections served as subjects for tracking TIL-B cells. As among solid tumors, invasive breast carcinomas with medullary features are of particular interest in respect to TIL-B cells, and we used those as positive controls. Immunohistology with Haematoxilin & Eosin showed a high level of tumor cell infiltration in the majority of the paraffin-embedded tissue sections in both solid tumors. IHC with CD20cy monoclonal antibody represented variable to considerable B cell infiltration in the investigated numerous tissue sections (Figure 1). Due to the substantial B cell infiltration found in invasive breast carcinoma with medullary features and metastatic melanomas, our complex strategy involving immunologic, molecular genetics, and biotechnological methods could be properly executed (Figure 2). By processing cancerous tissue specimen according to the methodological steps described in our Flowchart, we could successfully approach the questions on TIL-B cells. Tissue sections, cultured cancerous cells, and immune B cells found in the tumor microenvironment were all subjects of a next methodological pathway. Small pictures in the Flowchart show two essential milestones we've reached, demonstrating that the technology works. Fresh primary cell cultures of melanomas react well with specific anti-tumor antibody fragments of TIL-B origin.

### Heavy and Light Chain Immunoglobulin Variable Region Genes Could be Amplified and Cloned for Comparative DNA Sequence Analysis and scFv Antibody Fragment Construction

**Figure 3:**
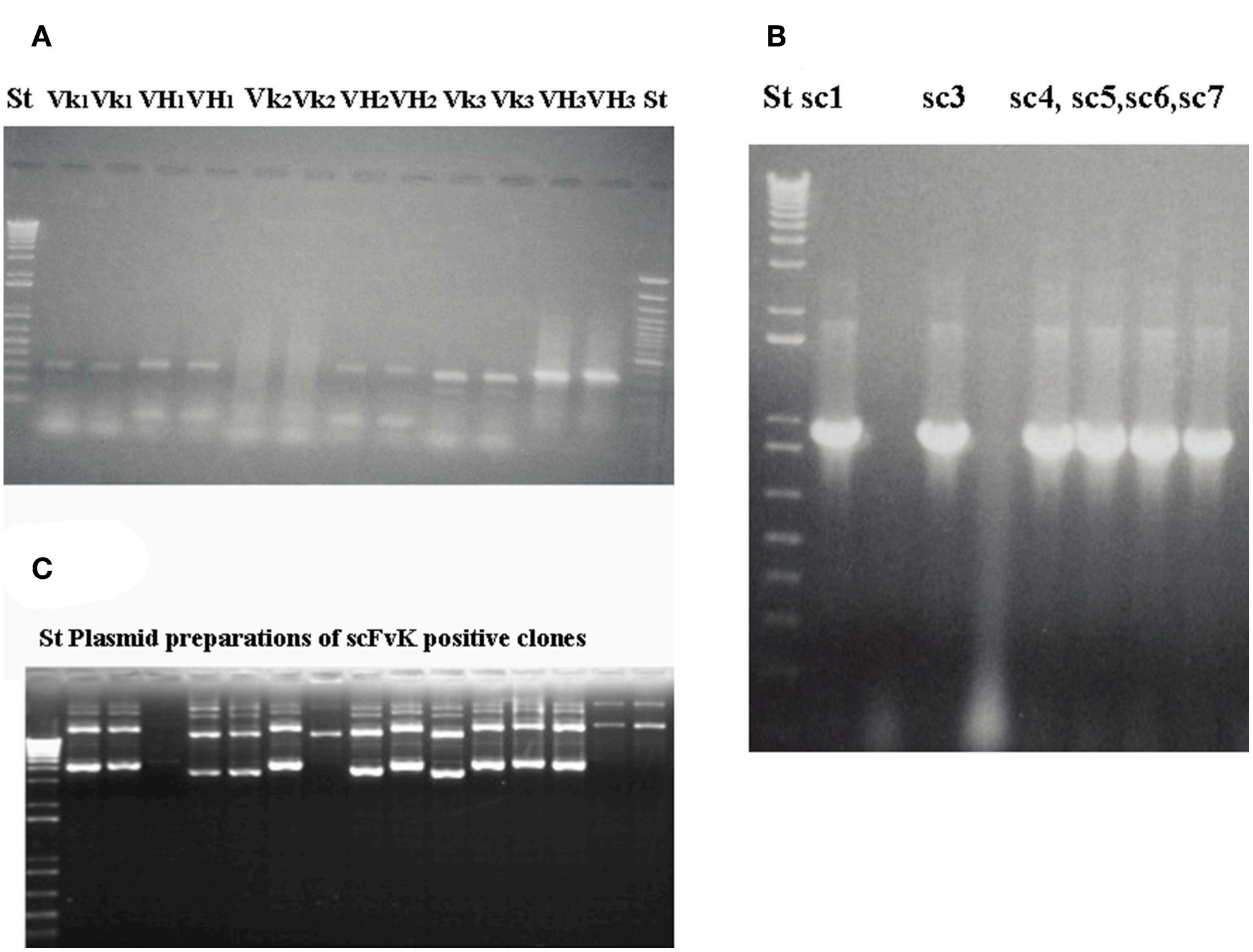
Gelelectrophoresis shows amplified immunoglobulin variable heavy and light chain gene regions and the constructed scFv antibody fragment. Preparative gel electrophoresis picture shows representative results with two parallels of amplified immunoglobulin VH and Vκ gene regions **(A)** from malignant melanoma cases (*n* = 3). Two standard marker lanes on the left and the right side help to identify the DNA Ig variable gene regions with their expected size (VH: 360 bp, Vκ: 340 bp) that would serve for the scFvK gene construction in the course of three step PCR reactions and purification. ScFvK was ligated into the phagemid vector for bacterial transformation and library generation. Molecular standards show the approximate sizes (800 bp) of the scFvK gene construct in the inserted clones. **(A)** and **(B)** are reproduced with permission from (22). QIAPrep plasmid minipreps of ScFvK inserted bacterial clones are ready for further analysis in terms of tumor binding **(C)**.

**Figure 4:**
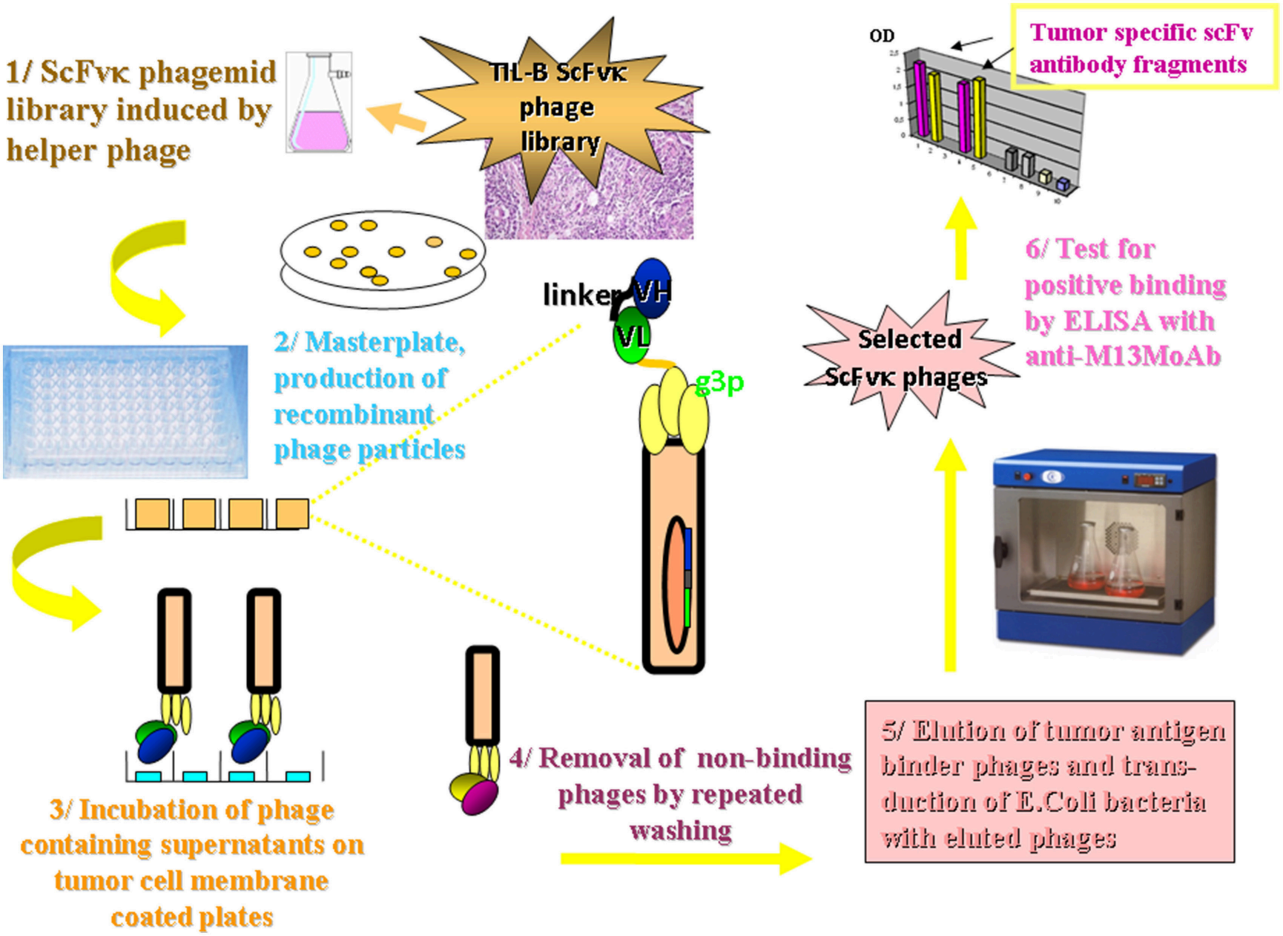
Methodology flow chart on the selection of tumor binder scFv fragments by ELISA. The flow chart presents the new tumor infiltrating B cell antibody fragment phage display technology. It describes the biotechnological processes and our detection system. A master plate was prepared that enabled the detection of our scFvK phage display library in a soluble scFv and a phage displayed scFv form after rounds of panning reactions. Antibody fragments could be selected in the course of an ELISA using 95 well maxisorbe plates precoated with native cancer membrane preparations.

**Figure 5:**
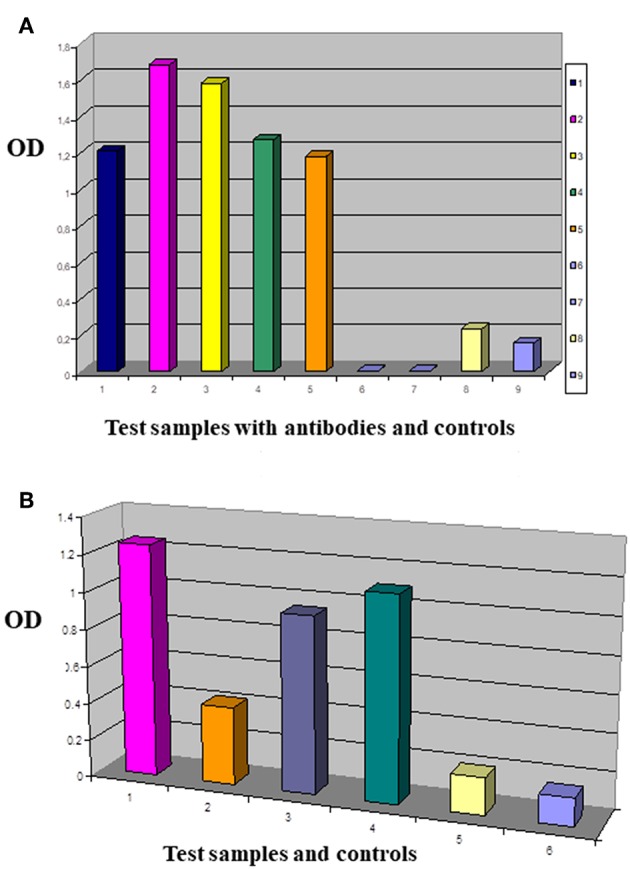
Comparing the tumor membrane binding capacity of antibody fragments. This is an enzyme labeled Immunosorbent Assay performed with test antibodies and antibody fragments in Nunc MaxiSorp® flat-bottom 96 well plates precoated with native tumor cell membrane preparations. IDC breast primary tumor cell culture membrane coated plates were reacted with antibodies of interest **(A)**: 1/MDA MB-231 panned sol scFv library (dark blue), 2/G2 sol scFvK (purple), 3/B2 sol scFvK (light green), 4/anti mucin antibody fragment (green), 5/HCBC3 anti GD3 antibody (orange), 8/medium control (yellow), 9/test/PBS background control (light blue). In the ELISA blocking assay two antibodies were used subsequently in one reaction **(B)**: 1/G2 scFv (purple), 2/HCBC3 (anti GD3) and G2scFv (orange), 3/HCBD1 (anti GD2) and G2scFv (violet), 4/IgG and G2scFv (dark green), 5/medium control (yellow), 6/background control with PBS (light blue). Optical density (OD) was measured at λ: 405 nm when p-Nitrophenyl Phosphate substrate was added after using alkaline phosphatase conjugated antibody.

Amplified melanoma TIL-B heavy and light chain immunoglobulin variable region gene PCR products were successfully cloned with an appropriate vector and bacterial transformation system. QiaGuick Qiagen plasmid preparations were qualified for a subsequent DNA sequence analysis. Ig variable region heavy and light chain DNA region inserted bacterial clone plasmid preparations were subjects for subsequent three-step PCR amplification, according to previously used techniques (19) for the scFvK antibody fragment construction (Figure 3). ScFVK antibody fragment phagemid library (9 × 10^10^ member size), generated with vector system (pHEN1, pCANTAB) was suitable for further testing. We could improve our phagemid ELISA tumor binder antibody selection technique with the usage of native cancer membrane fractions, obtained from melanoma primary cell cultures (Figure 4). The technology is suitable to define cancer binder scFv antibody fragments in their soluble form or as a protein expressed on the phage. Soluble scFv ELISA proved to be a reliable test system to analyze the characteristic tumor cell binding potential of the selected scFv antibody fragments (Figure 5). The technology is appropriate to compare the binding efficiency of antibodies of different origin and check for eventual cross-reactivity. Anti GD3 ganglioside binding capacity of our selected antibody fragments could be strengthened and further characterized by blocking ELISA experiments.

**Figure 6:**
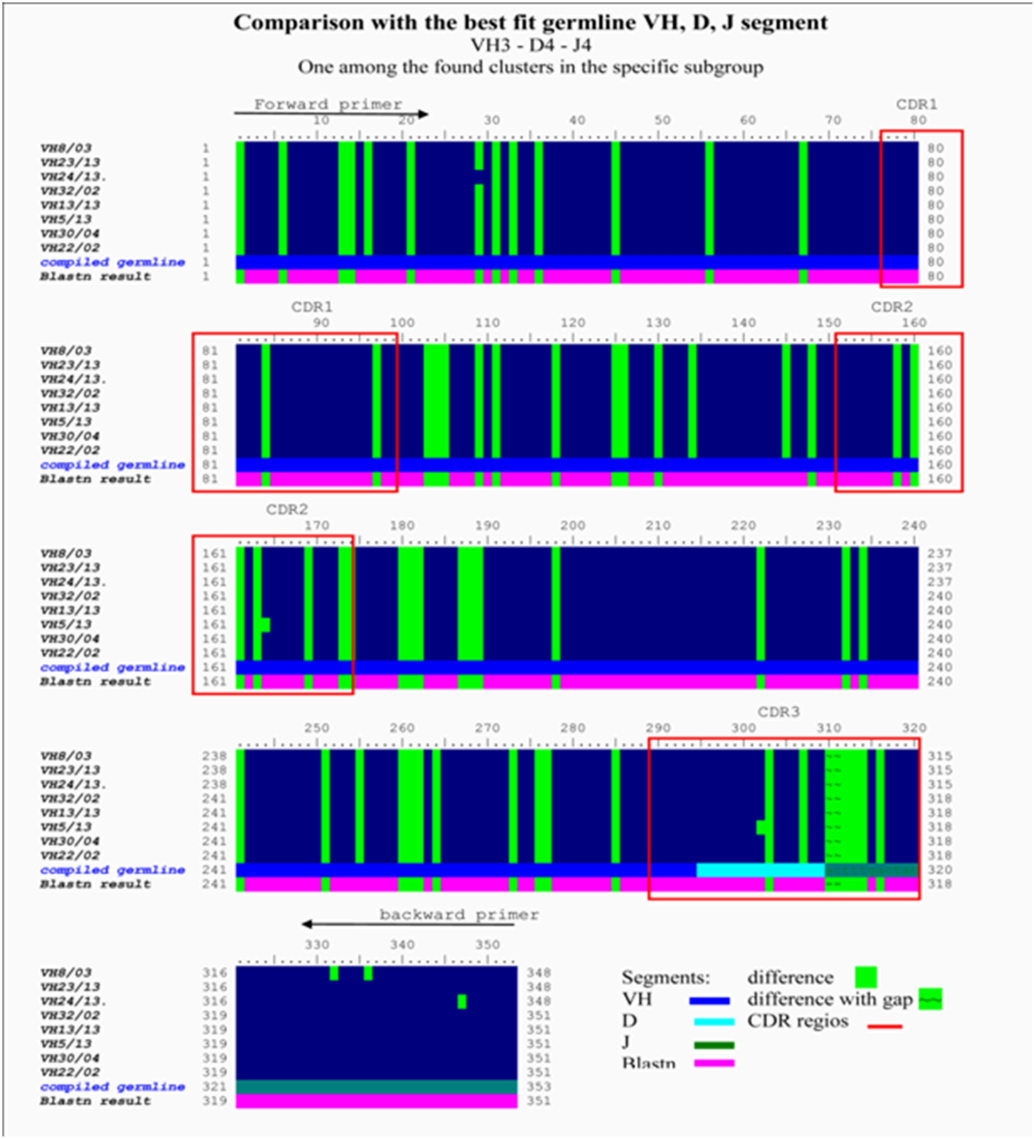
Homology ribbon representing comparative DNA sequence analysis results in the representative VH3 No I cluster. This is a comparison block that summarizes the alignment of previously defined, overrepresented Ig VH3 family cluster NoI immunoglobulin-variable region genes from invasive breast carcinoma with medullary features, defining the consensus DNA sequence of interest. Comparative DNA sequence analysis was performed with BioEDit, VectorNTI 11, and relevant databases (IMGT, Blastn) were used. Framework (FR1, FR2, FR3, FR4) and complementary determining (CDR1, CR2, CDR3) (red bordered squares) DNA regions show extremely high homology in this overrepresented cluster. Comparison with the best fit germline VH, D, J segment in the VH3/I subgroup was performed. Difference to compiled germline is shown with green label, while alteration found to blastn results are depicted with a purple lane. Consensus sequences defined in this homology ribbon alignment serve as control in the melanoma antibody repertoire analysis.

**Figure 7:**
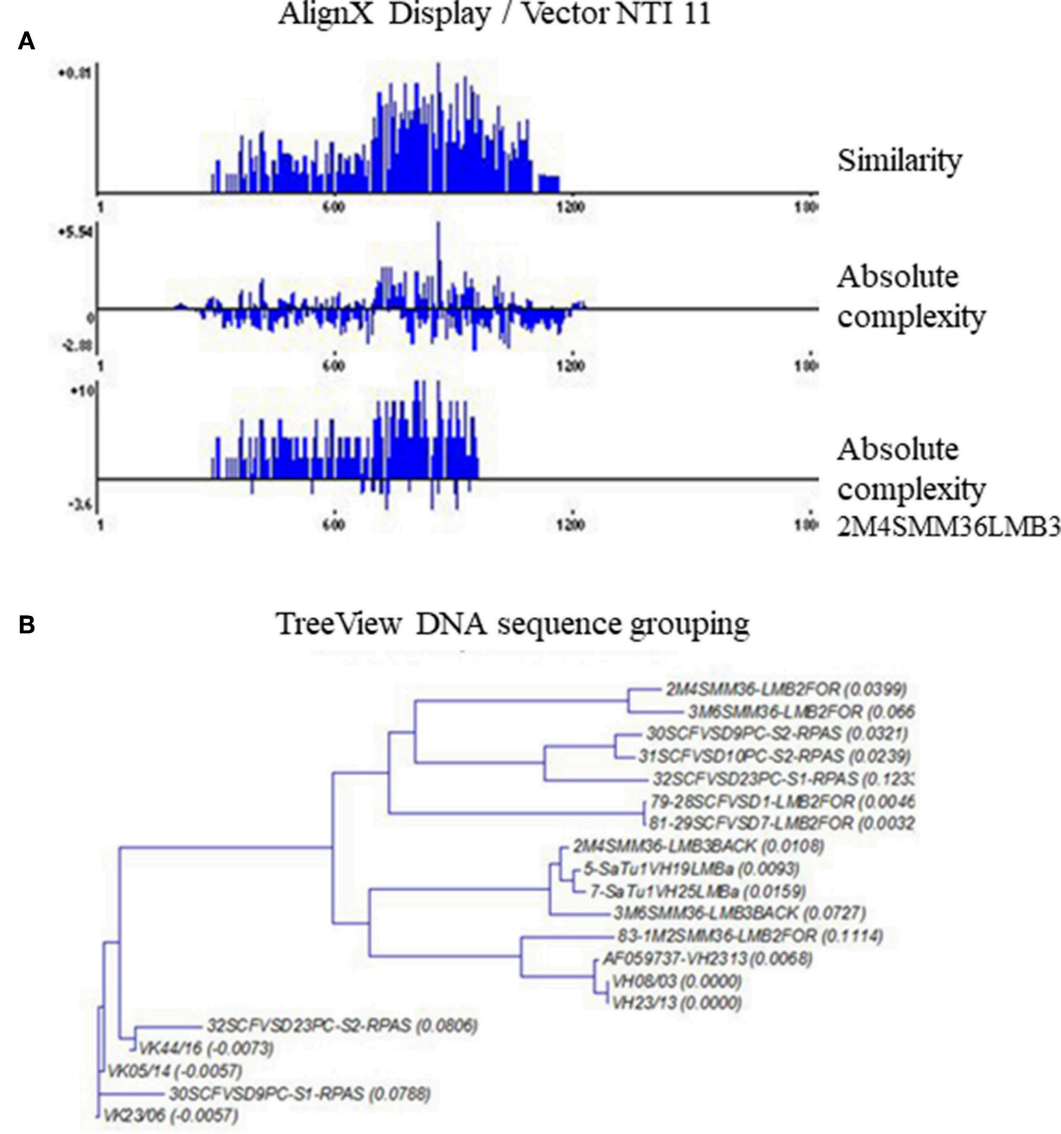
TreeView image to identify the aligned immunoglobulin variable gene regions highest similarity level. Cloned and randomly selected antibody variable region sequences in melanoma were subject to a detailed comparative DNA analysis by Clustal W, TreeView, and Vector NTI 11, in order to reveal DNA sequences with high homology to consensus. The levels of matches and mismatches in different parts of the aligned DNA sequences are indicated **(A)**. TreeView image shows the representative results, where melanoma tissue originated antibody variable region gene inserted clones represented substantial similarities to the previously defined anti GD3 antibody variable heavy chain sequences **(B)**.

A comparative DNA sequence analysis was performed and carefully evaluated by various DNA sequence analysis softwares and databases, as specified in the Materials and Methods section. We examined sequences from different melanoma clones but made comparisons with our previously defined disialylated GSL-specific antibody variable region coding DNA sequences, obtained from invasive breast carcinoma with medullary features. The present DNA analysis was compared to previously established essential comparative DNA analysis, indicated by the homology ribbon (Figure 6). Sequences belonging to the defined clusters were grouped into a tree structure by TreeView Analysis. We could identify members of the overrepresented VH3/1 cluster. The tendency for how the nearest VH3 sequences build groups is depicted in Figures 7A,B. Data show that among melanoma TIL-B antibody variable region heavy chain expressed genes, DNA sequences with extremely high homology to human anti-GD3 ganglioside antibody fragment could be found. A comparative DNA sequence analysis, on expressed tumor infiltrating B cell antibody fragment variable region genes, revealed highly homologous (98%) sequences to previously confirmed unique GD3 ganglioside binders. These data summarized first results of the comparative DNA sequence analysis, however, a more detailed DNA sequence analysis performed with Vector NTI advanced will be available shortly in an invited manuscript we are going to submit to *Immunome Research*.

### Evidence of Strong Disialylated Glycosphingolipid Expression on Primary Melanoma Cell Cultures and Established Cell Lines

**Figure 8:**
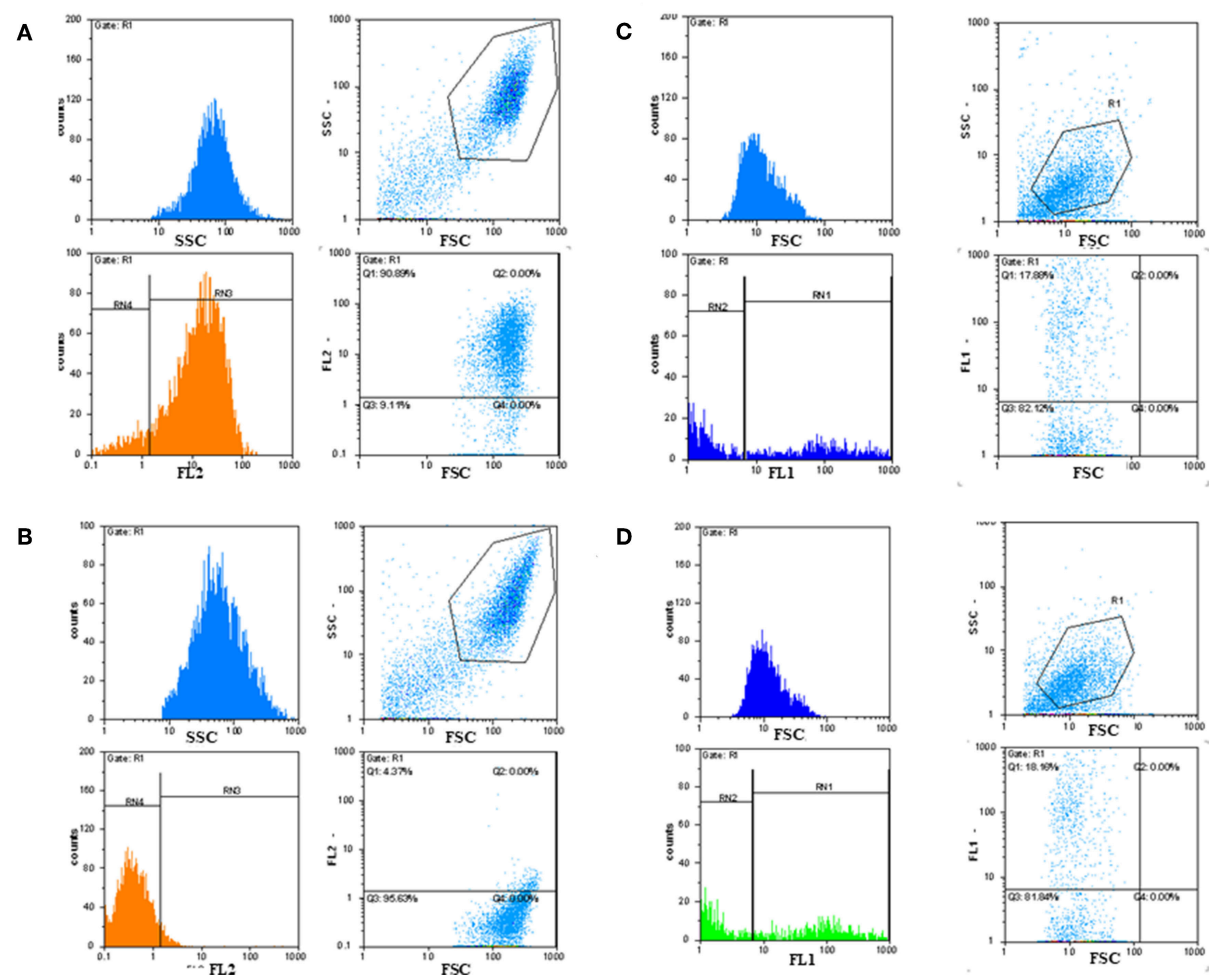
Detection of GD3 ganglioside positive cancerous cells by immunofluorescence FACS analysis. Indirect immunofluorescence assay and FACS analysis showed that tumor-associated disialylated glycosphingolipid specific antibodies reacted intensively with primary cancerous cell outgrowth from metastatic melanoma **(A)**, The majority (90%) of this cell population was GD3 ganglioside positive. Mean fluorescence intensity was about 100 times higher than the value of the negative control **(B)**. IF FACS analysis proved strong binding capacity of our scFv anti GD3 antibody fragments against the cultured A2058 melanoma cells **(C,D)**.

**Figure 9:**
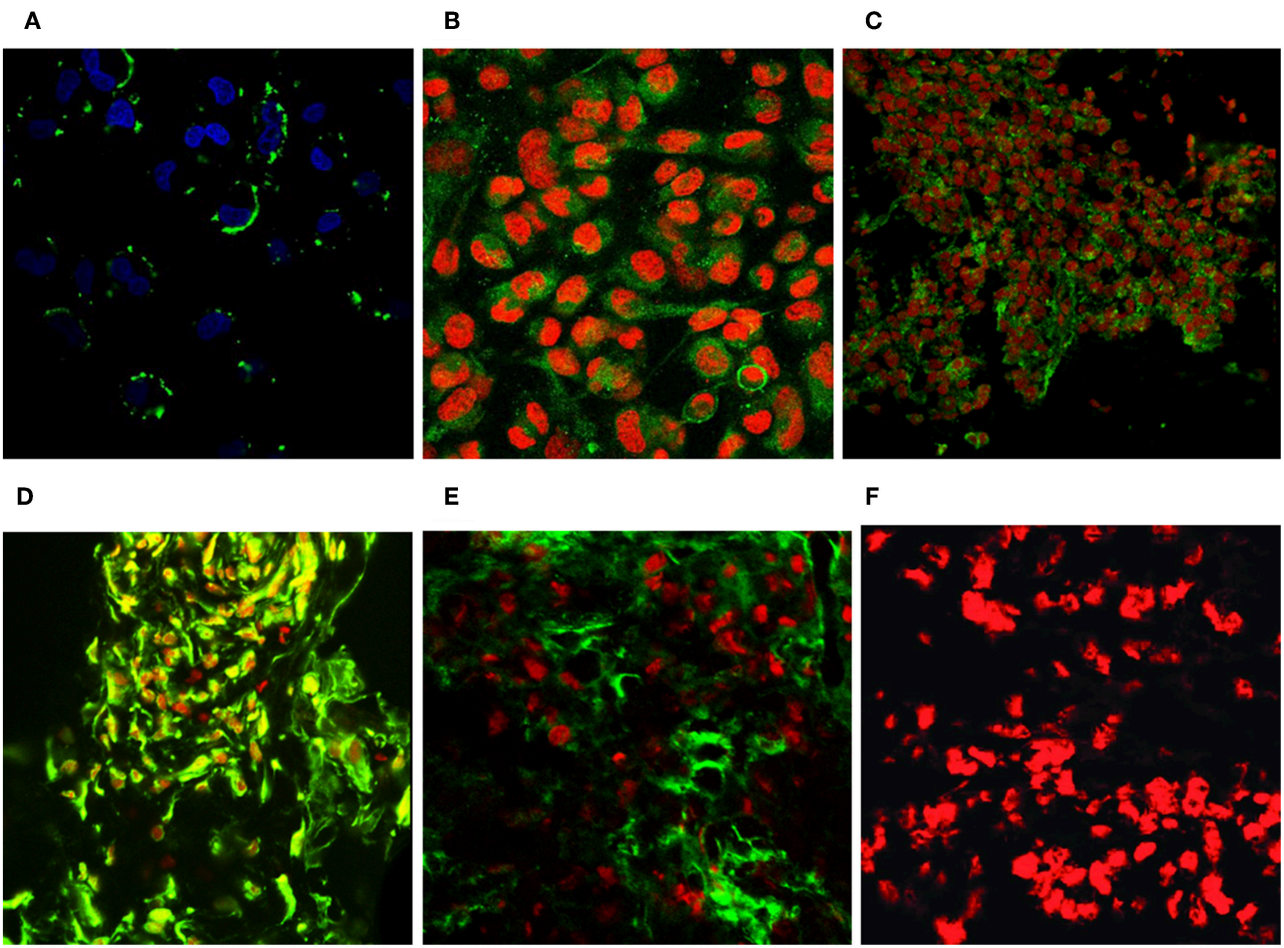
Revealing GD3 ganglioside expression on cultured melanoma cells and fresh frozen cancerous melanoma tissues. Unique GD3 ganglioside expression could be detected in chamber slide cultured melanoma cells [SK MEL28, M24, A2058 with the selected scFvK antibody fragment **(A–C)**]. **(C)** is reproduced with permission from (22). Fresh frozen melanoma tissue cryostat sections evidenced as well the positive reaction with our selected scFvK antibody fragment **(D)**. Commercially available antiGD3 ganglioside antibodies were also tested **(E)** on melanoma tissue sections and compared to the negative control **(F)**. FITC labeled anti-mouse IgG (Fab')2 and Anti-E Tag antibodies were used with propidium iodide or DAPI nucleus staining. The extinction and emission laser parameters for FITC detection were 488 and 520 nm, while for the propidium iodide they were 534 and 610 nm, respectively (200x, 400x).

Immunofluorescence FACS analysis with GD3-specific monoclonal antibodies could unambiguously define a strong expression of these unique tumor-associated antigens on primary melanoma cell cultures (Figure 8A). The great majority of the cell population showed significantly high immunofluorescence positivity, as compared to the background control values (Figure 8B). Mean fluorescence intensity was about 100 times higher than the value of the negative control. Results suggest the importance of these tumor-associated molecules for a strong cancer progression. These data strengthen our earlier findings, when fresh breast cancer cell lines were set up from invasive ductal carcinomas of the breast and used for testing tumor binder antibody fragments in the form of native membrane preparations. A considerable expression of disialylated GSLs could be shown with our selected soluble B2scFv and G2scFv antibody fragments on A2058 melanoma cell lines by indirect immunofluorescence FACS analysis (Figures 8C,D). Soluble preparations of these antibody fragments proved to be a good form for use of these molecules in immune assays like ELISA and immunofluorescence. Several other cell lines have been tested by us in the course of cell or cell membrane-based ELISA and IF-FACS (data will be shown elsewhere). Chamber slide cultures of SK-Mel 28, M24, and M2058 melanoma cell lines were set up. These cells showed strong disialylated GSL, that is GD3 ganglioside expression with our soluble scFv antibody fragments, in an immunofluorescence assay, visualized by confocal laser microscopy (Figures 9A–C). The present technique enabled the careful detection of GD3 gangliosides on melanoma cryostat tissue sections. Our selected scFvK antibody fragment gave an even stronger reaction with the disialylated GSLs (Figure 9D), when compared to the commercially available antibody (Figure 9E). Immunofluorescence labeling was validated by controls (Figure 9F). This is an important step forward, taking into consideration the difficulties and special care needed to detect GD3 gangliosides in general. so Detecting and also defining these highly tumor-associated GSLs is of potential diagnostic value, as they are functionally so very relevant to cancer progression and metastases.

### Novel Strategy for Harnessing the Host Immune System to Build New Cancer Diagnostics and Therapeutics

Our original theory could now be proved in melanoma. The methodological pathway, with immunological, molecular genetics, and biotechnological techniques is suitable to harness the cancerous tissue together with its tumor microenvironment for the search for cancer antigens. The acquired results can be used as cornerstones to reveal highly cancer-associated biomarkers. One unique impact of the strategy is that it can identify glycolipid-based structures, members of the glycosphingolipid family that play an essential role in cancerogenesis by their abnormal glycosylation. TIL-B cell derived scFvK disialylated GSL binder antibody fragments have the capacity to strongly label cancerous melanoma cells, as seen in the Flow chart (Figure 2). Our strategy and new data can now be further exploited with antibody engineering techniques to harness the tumor infiltrating B cell antibody repertoire to build novel diagnostics and immune-based therapeutics.

## Discussion

Our immunohistochemistry results support the presence of B cells in melanomas. The amount of these cells varies greatly, but the methodological Flow chart we developed is sensitive enough for the investigations. We can conclude that our complex new strategy, involving cellular immunological, molecular genetics, and biotechnological techniques processing the cancerous tissue itself, is suitable to approach the questions on TIL-B cell characteristics. This technology, with DNA analysis and TIL-B scFv antibody fragment phage display, enables the revealing of human antibody fragments with specificity to unique glycosylated residues in disialylated glycosphingolipids, GD3 gangliosides. This is an important finding, taking into consideration the difficulties that hindered detection of GSLs and that cancer cells are well characterized by aberrant glycosylation of the surface membrane (15, 21). Our original hypothesis that TIL-B cells would eventually have functions in terms of specific tumor-recognizing capacity in solid tumors, could be proved now in malignant melanoma. The study not only strengthened the hypothesis but would serve as an additional source, besides peripheral blood and lymph nodes, to obtain tumor-binder antibodies of human origin. We emphasize the importance of antibody repertoire analysis at the DNA level, in order to better understand the precise nature of natural human antibodies. Our results suggest that scFv antibody fragment construction based on heavy and light chain immunoglobulin variable region genes, originated from B cells in melanoma, provide a new source for antibody fragments with binding capacity to glycolipid and glycoprotein-based tumor-associated antigens. Our results presented here, evidencing the strong disialylated glycosphingolipid expression on primary melanoma cell cultures and established cell lines, underline the importance of the defined human GD3 ganglioside specific antibody fragments. As the detection of these disialylated glycosphingolipids on malignant cells and tissues have been challenging, the present findings serve as a solution to that problem. The special functional characteristics of these key disialylated glycosphingolipid molecules provides promise of a further diagnostic and/or therapeutic usage (23, 24).

Ongoing investigations show how the tumor changes its environment to modify and impair functions of the host immune apparatus. In addition to some well documented mechanisms, there are essential results available to support the hypothesis on shedding of certain tumor-associated GSLs (25). GSLs that shed from cancerous cells may serve to protect tumor cells from host immune destruction. Certain sialic acid-containing GSLs (gangliosides) have potent immune regulatory properties: inhibitory effects on antibody production *in vivo*, on the generation of antibody-synthesizing cells *in vitro* (26) and on lymphocyte proliferative responses to mitogens and antigens *in vitro* (27). Both bovine and human brain gangliosides, and gangliosides shed by antigen-stimulated lymphocytes (27) were shown to exhibit such immune regulatory properties.

As tumor-derived gangliosides affect immune cell functions and reduce the B lymphocytes' antibody production, we suspect an important B lymphocyte and cancer cell crosstalk mechanism. In order to reveal the cornerstone molecules of that regulation mechanism, the molecularly processed TIL-B cells and tumor-associated glycosphingolipid containing cancerous tissues are subject to a subsequent high-throughput gene expression data analysis.

Our finding, that TIL-B cells produce antibodies that are specific to tumor-associated disialylated glycosphingolipids, is of special interest, taking into consideration the importance of these molecules in tumor progression, invasion, metastases, and signal transduction (28, 29). We postulate that TIL-B cells, by producing unique anti-GD3 disialoganglioside-specific antibodies in the tumor-microenvironment serve a potential anti-tumor and an immunological regulation mechanism. Our novel tumor immunological protocol involving TIL-B antibody phage display and immunoglobulin profile analysis (30) to define the sialylated glycosphingolipid-binding capacity in a broader group of patients with metastatic melanoma is under evaluation. Antibodies with binding capacity to sialylated glycosphingolipid structures can neutralize shed gangliosides. This helps to restore the antitumor reaction that is diminished by shed tumor-associated molecules (31) and might influence the strength of the immune response in general.

We wonder, how far cancer cell editing is influenced by GSLs? It is well known that variation in sialylated glycosphingolipid composition, changes in membrane and/or cytoplasmic localization and the intensity of tumor antigen shedding into the microenvironment are important factors (32). Dramatic changes in glycolipid composition and metabolism were observed in spontaneous tumors, in addition to virally or chemically transformed cells (33, 34). Present data urge extensive immunological and biochemical analysis of sialylated glycosphingolipids, and further understanding of TIL-B cells' potential “crosstalk” in relation to cancer cells.

Interestingly, GD3 ganglioside directed monoclonal antibodies can bind to themselves and to each other. It is expressed within the VH region of anti-GD3 monoclonal antibodies, while the homophilic binding epitope must be bound to a surface. Homophilic binding may strongly contribute to the apparent avidity of mouse anti-GD3 for GD3 ganglioside. Shared or cross reactive idiotype may increase the avidity of antigen binding (35, 36). A subset of antibodies which carry an internal image of their own antigens would play an important role in the regulation of the humoral immune response through an idiotype network. The potential use of anti-idiotypic antibodies for cancer treatment continues to be a challenge for tumor immunotherapy. Failures and hopes obtained, with ganglioside mimicking anti-idiotypic antibodies and the existence of a natural response against gangliosides, suggest that these glycolipids could be idiotypically relevant antigens (37, 38). Our present results help to better understand this essential field. They provide relevant TIL-B Ig VH genes for further studies. Anti-idiotypic antibodies as cancer vaccines, with achievements and possible future improvements, have always been an expectant area of interest (39–41).

According to the above findings, TIL-B cells' unique GD3 ganglioside-specific antibody producing capacity offers a potential immune regulatory mechanism in the tumor-microenvironment. These results show novel interactions between the immune components of the tumor microenvironment and the cancer cells. This potential tumor-immunological regulation mechanism would complete existing theories, summarized on galectin-glycan interactions and B cells (42).

Antibodies to disialylated GSLs can neutralize shed gangliosides and thus help to restore the diminished T and B cell functions, caused by GSLs. However, as only a few antigens have been identified by TIL-B cells (43–46) our TIL-B disialylated ganglioside binder antibody fragments could be a real asset. Based on the results, we can conclude that TIL-B cells‘ immunoglobulin variable region gene usage with unique disialylated glycosphingolipid revealing capacity is a subject to be harnessed by further antibody engineering, in order to develop cancer targeting tools. Consequently, present data would lead to new early diagnostics and innovative immune-based cancer therapeutics.

## Ethics Statement

This study was carried out in accordance with the recommendations of Bioethic Codex, Principles and practice of medical and biological/clinical research (2016. január) (https://ett.aeek.hu/bioetikai-kodex/ guidelines (that was written and approved by Board of Directors of Medical Research Council, Hungary), with written informed consent from all subjects. All subjects gave written informed consent in accordance with the Declaration of Helsinki. The protocol was approved by the Medical Research Council, Hungary, coding number of the Ethical permission we received for our project: (ETT TUKEB 16462-02/2010, 336/2014.9710-1/2015/EKU).

## Author Contributions

BK was responsible for the main idea, new methodology development, realization of preparative and experimental works, and wrote the manuscript. SH took minor cancerous tissue samples. KE took punch biopsies from cancerous tissues. VP carried out microscopic evaluation of histology and immunohistochemistry. GN summarized and updated patients' history data. KC, working in melanoma patient care, dealt with patients' formal consent, as well as minor cancerous tissue and blood sampling. MB, an experienced scientist in immunology, gave methodological suggestions in high-precision processes. EF, Head Surgeon in our Institute, arranged for access to the surgical materials. LT, Head Surgeon in our Institute, arranged necessary ethical permission issues. JT gave technical help in immunofluorescence confocal laser microscopy. AS helped with scientific technical issues, methodological obstacles, manuscript editing. YS, an experienced clinician scientist in immunological disorders, helped evaluating natural human antibody data. MG has strong experience in image analysis in radiological diagnostics and detects cancer metastases. As former director of the National Institute of Oncology, MK's suggestions and arrangements enabled to expand this tumor immunological project into a further melanoma patient follow-up study. GL, an experienced clinician, Head of the Oncoteam and Melanoma Patient Care Unit, arranged for innovative treatment strategies for melanoma patients and reported according to our Ethical Permission.

### Conflict of Interest Statement

The authors declare that the research was conducted in the absence of any commercial or financial relationships that could be construed as a potential conflict of interest.

## Abbreviations

AP: alkaline phosphatase
ATCC: american type cell collections
AEC: 3-amino-9-ethylcarbazole (substrate)
BSA: bovine serum albumin
CSC: cancer stem cells
GD3 ganglioside type: disialylated glycosphingolipids
ELISA: enzyme labeled immunosorbent assay
FBS: fetal bovine sera
FITC: fluorescein isothiocyanate
FDA: food and drug administration
GSL: glycosphingolipid
H&E: haematoxilin & eosin
HJLCT: Harry J Loyd Charitable Trust
HSA: human serum albumin
HRP: hydrogen peroxide
IF: immunofluorescence
Ig: immunoglobulin
IHC: immunohistochemistry; Ministry of Human Capacities in Hungary, hungarian medical research council
NM: nodular melanoma
O'AcGD3: O'-acetylated GD3
PFA: paraformaldehyde
PBMC: peripheral blood mononuclear cells
PBS: phosphate buffered saline
PNPP: p-Nitrophenyl Phosphate
PCR: polymerase chain reaction
scFv antibody: single-chain-variable antibody
SSM: superficial spreading melanoma
TIL-B: tumor infiltrating B cells.
